# TdIF1-LSD1 Axis Regulates Epithelial—Mesenchymal Transition and Metastasis via Histone Demethylation of E-Cadherin Promoter in Lung Cancer

**DOI:** 10.3390/ijms23010250

**Published:** 2021-12-27

**Authors:** Qi Liu, Juan Xiong, Derong Xu, Nan Hao, Yujuan Zhang, Yi Sang, Zhigang Wang, Xiufen Zheng, Jeffrey Min, Hong Diao, Jacques Raphael, Saman Maleki Vareki, James Koropatnick, Weiping Min

**Affiliations:** 1 Institute of Immunotherapy, College of Basic Medicine, The First Affiliated Hospital of Nanchang University, Jiangxi Academy of Medical Sciences, Nanchang 330046, China; qliu442@uwo.ca (Q.L.); xudr@ncu.edu.cn (D.X.); 18291009428@163.com (N.H.); yujuanzhang@ncu.edu.cn (Y.Z.); sangyi10@foxmail.com (Y.S.); wzg2700@sina.com (Z.W.); 2 Department of Pathology and Laboratory Medicine, University of Western Ontario, London, ON N6A 5A5, Canada; xzheng26@uwo.ca (X.Z.); Saman.MalekiVareki@lhsc.on.ca (S.M.V.); jkoropatnick@gmail.com (J.K.); 3 Department of Preventive Medicine, School of Medicine, Shenzhen University, Shenzhen 518054, China; jxiong@szu.edu.cn; 4 Department of Surgery, University of Western Ontario, London, ON N6A 5A5, Canada; 5 Department of Microbiology and Immunology, University of Western Ontario, London, ON N6A 5A5, Canada; 6 Department of Oncology, University of Western Ontario, London, ON N6A 5A5, Canada; jacques.raphael@lhsc.on.ca; 7 London Regional Cancer Program, Matthew Mailing Centre for Translational Transplantation Studies, Lawson Health Research Institute, London, ON N6A 5A5, Canada; jmin58@uwo.ca (J.M.); hdiao2@uwo.ca (H.D.)

**Keywords:** TdIF1, EMT, LSD1, NSCLC

## Abstract

We have previously found that TdT-interacting factor 1 (TdIF1) is a potential oncogene expressed in non-small cell lung cancer (NSCLC) and is associated with poor prognosis. However, its exact mechanism is still unclear. The lysine-specific demethylase 1 (LSD1) is a crucial mediator of the epithelial–mesenchymal transition (EMT), an important process triggered during cancer metastasis. Here, we confirm that TdIF1 is highly expressed in NSCLC and related to lymph node metastasis through The Cancer Genome Atlas (TCGA) analysis of clinical samples. Silencing TdIF1 can regulate the expression of EMT-related factors and impair the migration and invasion ability of cancer cells in vitro. An analysis of tumor xenografts in nude mice confirmed that silencing TdIF1 inhibits tumor growth. Furthermore, we determined the interaction between TdIF1 and LSD1 using immunoprecipitation. Chromatin immunoprecipitation (ChIP) revealed that TdIF1 was enriched in the E-cadherin promoter region. The knockdown of TdIF1 repressed the enrichment of LSD1 at the E-cadherin promoter region, thereby regulating the level of promoter histone methylation and modulating E-cadherin transcription activity, ultimately leading to changes in EMT factors and cancer cell migration and invasion ability. The LSD1 inhibitor and TdIF1 knockdown combination showed a synergistic effect in inhibiting the growth, migration, and invasion of NSCLC cells. Taken together, this is the first demonstration that TdIF1 regulates E-cadherin transcription by recruiting LSD1 to the promoter region, thereby promoting EMT and tumor metastasis and highlighting the potential of TdIF1 as a therapeutic target for NSCLC.

## 1. Introduction

Lung cancer is the most common malignant cancer and the leading cause of cancer-related deaths in both men and women worldwide [1]. Non-small cell lung cancer (NSCLC) comprises more than 80% of all lung cancer cases, and its main subtypes include lung squamous cell carcinoma (LUSC) and lung adenocarcinoma (LUAD) [2,3]. Treatments for NSCLC include surgical resection, radiotherapy, chemotherapy, immunotherapy, and targeted therapies. Although relevant treatment research has made significant progress in recent years, the five-year overall survival rate of NSCLC patients remains less than 20% [4]. Various factors affect NSCLC development, progression, and patient outcome, including mutation, metastasis, and drug resistance [5,6,7]. However, we do not yet have a complete understanding of the mechanism. Therefore, further studies of NSCLC-related molecular mechanisms are essential to improve treatments and the survival rates of NSCLC patients.

TdT-interacting factor 1 (TdIF1), also known as deoxynucleotidyl transferase terminal interacting protein 1 (DNTTIP1), is a DNA-binding protein homologous to p65/NF-κB. It regulates V(D)J recombination via interaction with terminal deoxynucleotidyl transferase (TdT) [8]. Studies have found that TdIF1 can directly bind to the transcription factor TRERF1 (TRep-132); therefore, it can be involved during transcriptional regulation [9]. We have previously found that TdIF1 is highly expressed in NSCLC, and it is associated with poor patient prognosis [10]. However, the exact mechanism underlying TdIF1-mediated transcriptional regulation in cancer cells remains unclear.

Epithelial-to-mesenchymal transition (EMT) is a reversible biological process in which cells undergo a transition from an epithelial phenotype to a mesenchymal state. Cancer cells undergoing EMT can gain greater invasiveness, becoming more aggressive and entering the surrounding stroma, thereby changing the tumor microenvironment and making it conducive to cancer progression and metastasis [11]. Moreover, EMT has been associated with acquired EGFR-TKI resistance in NSCLC. However, the mechanism underlying EMT-induced NSCLC progression and drug resistance has not been fully explored [12]. We have previously revealed that the higher expression of TdIF1 is correlated with metastasis in NSCLC [10]. However, the mechanism by which TdIF1 regulates EMT has never been reported.

Lysine-specific demethylase 1 (LSD1/KDM1A/AOF2) was the first histone demethylase to be discovered. Its primary function is to specifically demethylate mono- and di-methylated histone H3 lysine 4 (H3K4) and histone H3 lysine 9 (H3K9) [13]. It is often overexpressed in NSCLC and promotes proliferation and invasion [14]. Thus, epigenetic change is an essential feature during the development of NSCLC and a viable target for the treatment of lung cancer. Currently, preclinical studies and clinical trials based on a variety of histone-modifying enzymes and their inhibitors are ongoing [15]. In a bioinformatic analysis of TdIF1-related interacting molecules, we found that TdIF1 may be related to lysine-specific demethylase 1 (LSD1), but the mechanism of action of TdIF1 in NSCLC progression remains unknown [10].

In this study, we demonstrate, for the first time, that TdIF1 regulates NSCLC cell EMT in vitro and in vivo and thus leads to cancer metastasis and poor prognosis. TdIF1 interacts with LSD1 and recruits LSD1 to the E-cadherin promoter, resulting in transcriptional inhibition due to nucleosome H3K4 demethylation. The knockdown of TdIF1 in NSCLC cell lines reduced EMT and tumor metastasis. Furthermore, the expression of exogenous TdIF1 enhanced the enrichment of LSD1 at E-cadherin gene promoters, induced histone demethylation, resulting in E-cadherin transcriptional repression, and increased migration and invasion. Moreover, the administration of an LSD1 inhibitor abolished TdIF1 overexpression, promoting cell migration and invasion. Thus, the LSD1 inhibitor and TdIF1 knockdown combination showed a synergistic effect in inhibiting the growth, migration, and invasion of NSCLC cells. Our findings suggest that TdIF1 regulates EMT via the histone demethylase LSD1, showing its potential as a novel and targeted therapy for NSCLC.

## 2. Results

### 2.1. TdIF1 Is Highly Expressed in NSCLC and Positively Correlated with Metastasis

**Figure 1:**
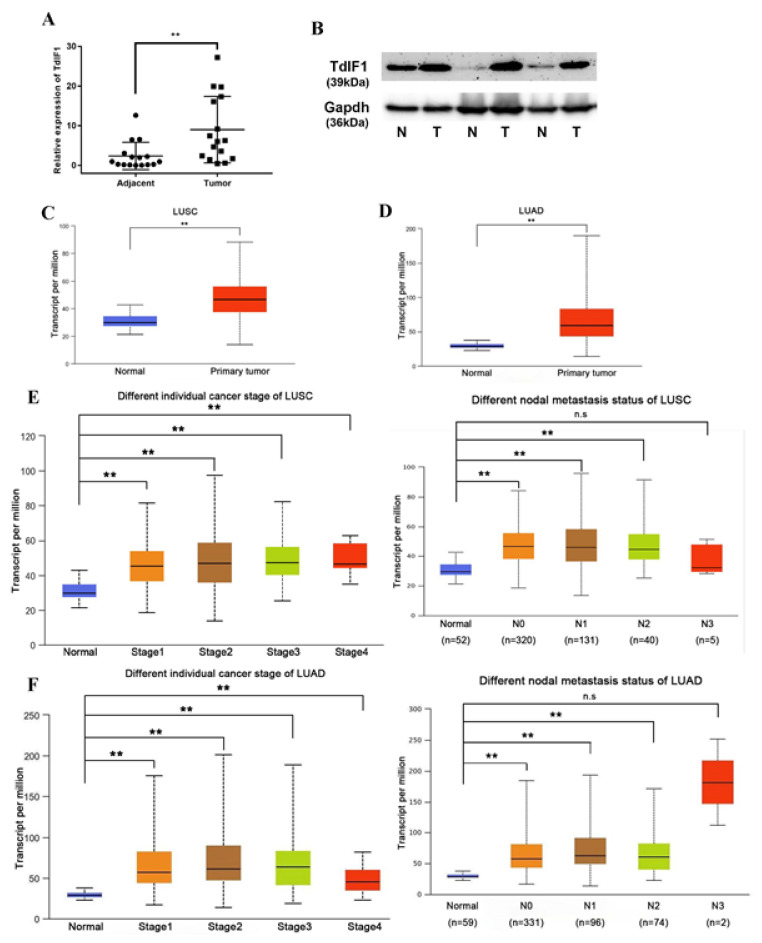
TdIF1 is highly expressed in NSCLC and positive relatively correlated with metastasis. (**A**,**B**) TdIF1 expression in NSCLC patient tissue samples. Tumor tissues (T) and paired adjacent normal tissues (N) were collected from patients and the expression level of TdIF1 was measured through qPCR (**A**) and Western blot analysis (**B**). (**C**–**F**) The Cancer Genome Atlas (TCGA)-based bioinformatic analysis of TdIF1 in NSCLC. The expression levels of TdIF1 in LUSC and normal tissues (**C**), and LUAD tissues and normal tissues (**D**) obtained from the TCGA database are displayed. The association of TdIF1 with different individual cancer stages and N stages in LUSC (**E**) and LUAD (**F**) are displayed. Data are shown as mean ± SD. ** *p* < 0.01.

To assess TdIF1 expression in NSCLC, we selected 16 pairs of tissue samples isolated from adenocarcinoma and squamous cell carcinoma patients. TdIF1 showed low expression in normal lung tissue but high expression levels in lung adenocarcinoma and squamous carcinoma (Figure 1A). Moreover, the freshly harvested lung cancer tissue specimens and paired healthy peripheral lung tissue specimens were subjected to Western blot analysis. TdIF1 protein expression in peripheral lung tissues was significantly lower than in lung cancer tissues (Figure 1B).

We previously reported that TdIF1 expression was positively correlated with N stage and lymph node metastasis, but not with the degree of differentiation and other clinicopathological factors [10]. To further establish the relationship between TdIF1 expression and clinicopathological factors, in silico analysis was conducted using TCGA database. These results demonstrated that the transcriptional level of TdIF1 is elevated in squamous cell carcinoma (Figure 1C) and lung adenocarcinoma (Figure 1D) compared to adjacent normal tissue. Furthermore, the expression level of TdIF1 was positively correlated with tumor stage and the extent of lymph node metastasis (Figure 1E,F).

### 2.2. Knockdown TdIF1 Inhibits the Migration and Invasion of Lung Cancer Cells

**Figure 2:**
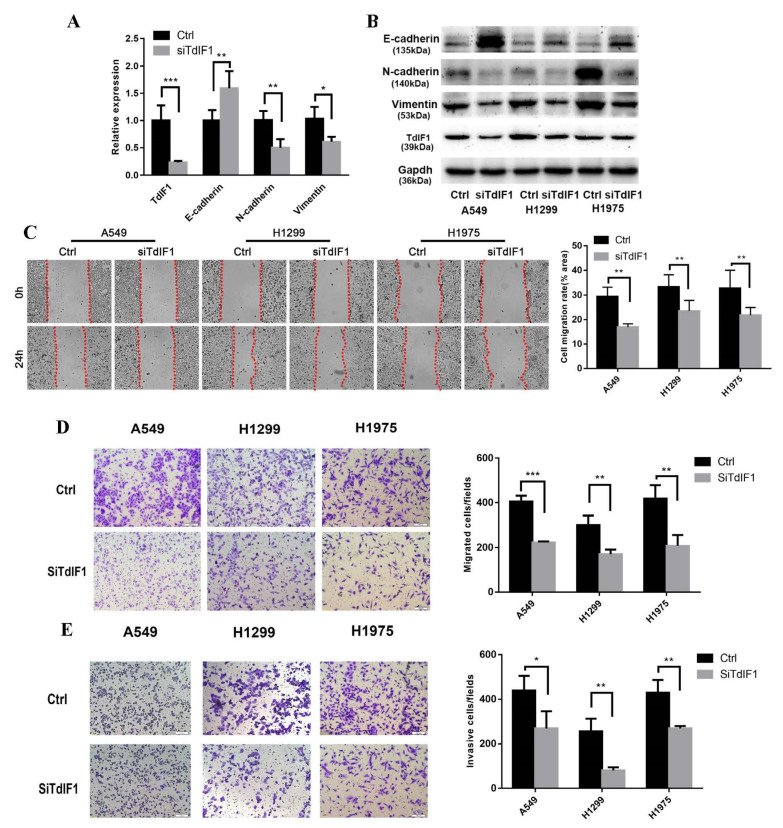
TdIF1 knockdown inhibits the migration and invasion of lung cancer cells. (**A**) Silencing TdIF1 regulates E-cadherin, N-cadherin, and vimentin expression level. A549 cells were transfected with TdIF1 siRNA(siTdIF1) and GL2 siRNA (Ctrl). The expression level of the indicated mRNAs was analyzed via qPCR. (**B**) TdIF1 regulates EMT-related proteins in NSCLC cells. After gene silencing with siRNA, the expression levels of indicated proteins were detected via Western blot analysis (**B**). (**C**–**E**) TdIF1 knockdown regulates the migration and invasion of NSCLC cells. Ctrl or siTdIF1-transfected NSCLC cells were subjected to wound healing (**C**), transwell migration (**D**), and Matrigel pre-coated transwell invasion (**E**) assays. Data are shown as mean ± SD. * *p* < 0.05, ** *p* < 0.01, and *** *p* < 0.001.

We previously found that TdIF1 was significantly upregulated in NSCLC and associated with the extent of lymph node metastasis [10]. Therefore, we measured EMT-related factors, E-cadherin, N-cadherin, and vimentin, in vitro after the knockdown of TdIF1 in three lung adenocarcinoma cell lines. After gene silencing TdIF1, the expression of E-cadherin was upregulated, while the expression of N-cadherin and vimentin was significantly repressed, as detected by the qPCR (Figure 2A). In support of TdIF1 involvement in EMT regulation, the silencing of TdIF1 also resulted in an increased E-cadherin level, and it downregulated the expression of N-cadherin and vimentin protein levels in A549, H1299, and H1975 cells (Figure 2B).

We next measured the TdIF1-regulated functional alterations of cell migration and invasion in NSCLC using wound-healing assays. After the knockdown of TdIF1, the migration ability of A549, H1299, and H1975 cells was inhibited (Figure 2C). Furthermore, we conducted transwell assays that confirmed that knockdown of TdIF1 significantly inhibited migration (Figure 2D) and invasion (Figure 2E) in A549, H1299, and H1975 cells.

### 2.3. Knockdown TdIF1 Inhibits NSCLC Cells Tumorigenesis In Vivo

**Figure 3:**
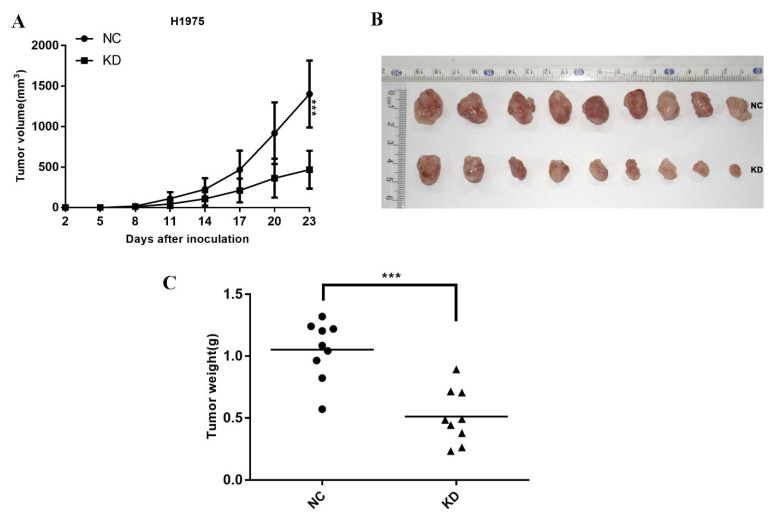
Knockdown of TdIF1 inhibits NSCLC carcinogenesis in vivo. (**A**–**C**). TdIF1 knockdown inhibits tumor growth. The control shRNA transfected (NC) and TdIF1-shRNA knocked down (KD) H1975 cells were injected subcutaneously to nude mice with 2 × 10^6^/mouse. The tumors were measured using vernier calipers every three days, and the tumor volumes were calculated by the formula V = (L × W^2^)/2 (**A**). At the endpoint of the experiment, the tumors were isolated and photted (**B**) and weighed (**C**). Data are shown as mean ± SD (*n* = 9). *** *p* < 0.001.

To explore the role of TdIF1 on tumorigenesis in vivo, we inoculated the H1975 cells stably transfected with either shTdIF1 or an empty vector into nude mice. The growth of the xenograft tumors after knocking down TdIF1 was significantly suppressed compared with the growth of tumors transfected with empty, non-targeting shRNA (Figure 3A). Furthermore, the tumors with knocked down TdIF1 were substantially smaller (Figure 3B) and lighter (Figure 3C) than those without TdIF1 knockdown. These data indicate that the knockdown of TdIF1 inhibits tumor growth and reduces the disease burden in vivo.

### 2.4. TdIF1 Interacts with LSD1 and Regulates EMT in NSCLC Cells

Our previous ingenuity pathway analysis (IPA) identified LSD1 as a potential interactor with the TdIF1 signaling network. LSD1 may also be involved in the histone methylation and epigenetic regulation of EMT and metastasis in NSCLC [10]. To determine whether TdIF1 can directly regulate LSD1, we transfected H1299 and H1975 cells with siTdIF1 and examined the expression of LSD1 via qPCR and Western blot analysis. Interestingly, there was no significant change in the levels of LSD1 between the control cells and TdIF1-knockdown cells (data not shown), suggesting that TdIF1 may not directly regulate the expression of LSD1.

**Figure 4:**
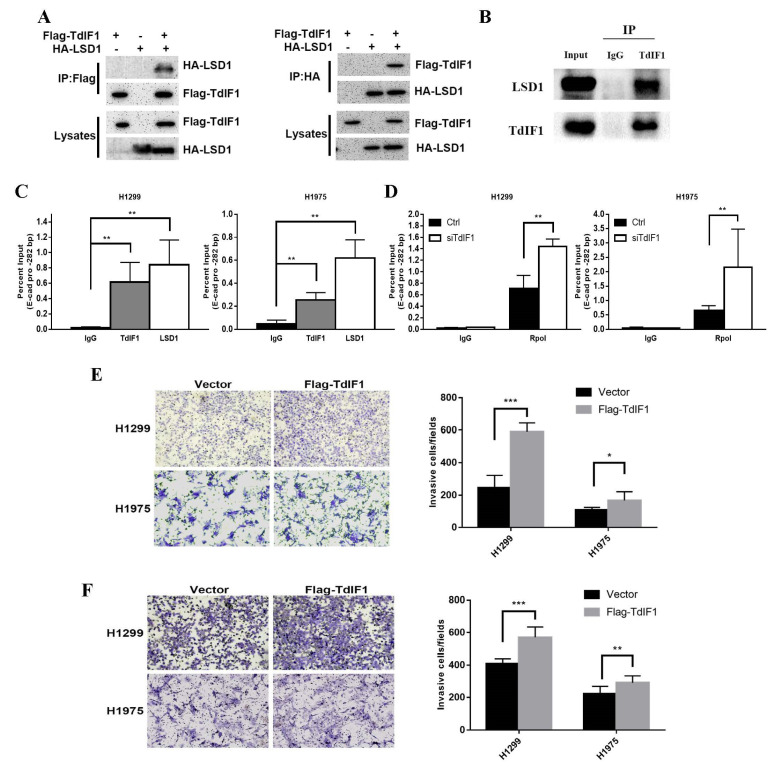
TdIF1 interacts with LSD1 and regulates NSCLC cell EMT. (**A**) Exogenous Co-IP of TdIF1 and LSD1. HEK293T cells were co-transfected with Flag-TdIF1 or HA-LSD1 as indicated. The clarified lysates from the cells were subjected to immunoprecipitation. After that, the precipitates were analyzed via immunoblotting. (**B**) Endogenous Co-IP of TdIF1 and LSD1. The co-IP assay with clarified lysates from A549 cells was performed as described in (**A**). (**C**) LSD1 and TdIF1 localize at the promoter region of E-cadherin. Crosslinked chromatin from H1299 and H1975 cells was isolated from the cells, followed by sonication to shear chromatin into DNA fragments. The DNA fragments were immunoprecipitated by antibodies as indicated. After purification, the enrichment of ChIPed DNA was detected via qPCR. Genomic region tests were as indicated. (**D**) TdIF1 regulates the binding of RNA Pol II at E-cadherin promoter. The enrichment of RNA Pol II in H1299 and H1975 cells transfected with siGL2 (Ctrl) and TdIF1 siRNA (siTdIF1) was measured using ChIP-qPCR as described in (**C**). (**E**,**F**) Migration and invasion ability of cells overexpressing TdIF1. The migration and invasion capacity of Flag-TdIF1 or empty vector-transfected H1299 and H1975 cells were measured as described in (Figure 2D,E). Data are shown as mean ± SD. * *p* < 0.05, ** *p* < 0.01, *** *p* < 0.001.

We then hypothesized that TdIF1 could influence the recruitment of LSD1, which might then contribute to the epigenetic modification of EMT. To validate whether TdIF1 may regulate EMT through LSD1 in NSCLC, we performed a co-IP assay to demonstrate the interaction between TdIF1 and LSD1. Our results show that HA-LSD1 was co-immunoprecipitated with Flag-TdIF1 in 293 cells (Figure 4A). Consistent with these results, the endogenous LSD1 protein could be immunoprecipitated with the TdIF1 antibody in A549 cells (Figure 4B), indicating that TdIF1 and LSD1 can form a protein complex in lung cancer cells.

We next investigated whether TdIF1 can bind to the E-cadherin promoter region using chromatin immunoprecipitation (ChIP) analysis. Chromatin from H1299 and H1975 cells was first immunoprecipitated with control immunoglobulin G, anti-TdIF1, and anti-LSD1 antibodies, and qPCR was performed on the recovered DNA to determine the enrichment at the E-cadherin proximal promoter regions. The occupancy of TdIF1 was detected significantly at the promoters of E-cadherin, as well as LSD1, but the IgG showed no occupancy (Figure 4C).

To confirm that E-cadherin is indeed inhibited by TdIF1, we carried out a similar ChIP assay to determine the binding of RNA polymerase II at the E-cadherin promoter. Consistent with E-cadherin expression, high levels of RNA polymerase II were detected at the E-cadherin promoter in TdIF1-KD H1299 and H1975 cells. In contrast, this binding was abolished mainly in control cells (Figure 4D).

Our previous data and analysis revealed that TdIF1 knockdown inhibited the migration and invasion of NSCLC cells (Figure 2). To further confirm that TdIF1 is functionally involved in promoting NSCLC cell migration and invasion, we generated TdIF1-overexpressing cells by transfecting Flag-TdIF1 plasmids into H1299 and H1975 cells. The results reveal that the overexpression of TdIF1 upregulated lung migration (Figure 4E) and invasion ability (Figure 4F) in vitro. Taken together, these data reveal that TdIF1 interacts with LSD1, binds to E-cadherin promoter, and regulates EMT as well as cell migration and invasion in NSCLC cells.

### 2.5. TdIF1 Regulates H3K4me2 Levels at the E-Cadherin Promoter by Recruiting LSD1

**Figure 5:**
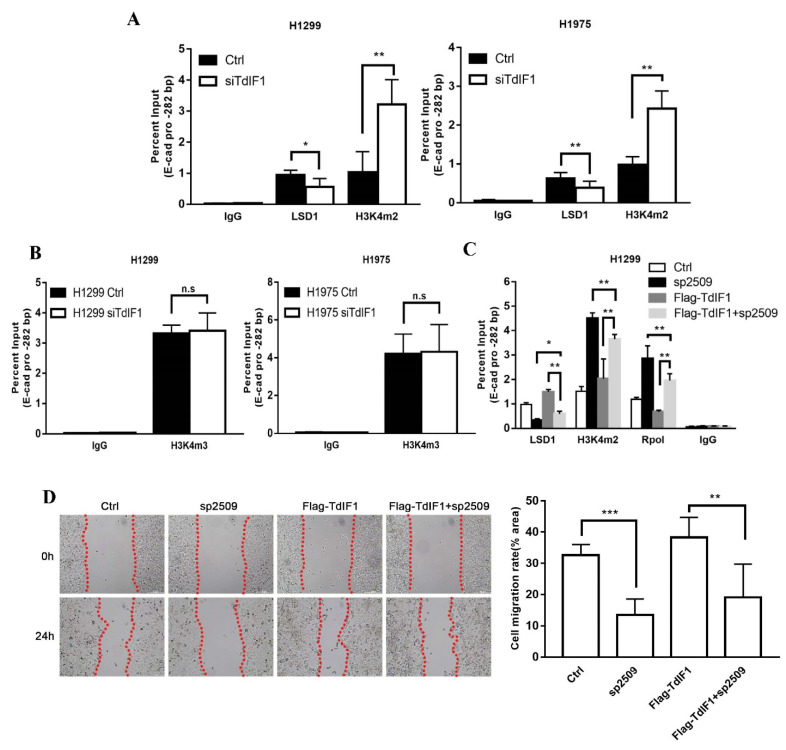
TdIF1 downregulates H3K4me2 levels at the E-cadherin promoter through the recruitment of LSD1. (**A**) TdIF1 regulates LSD1 and H3K4me2 enrichment at the E-cadherin promoter. The enrichment of LSD1 and H3K4m2 in siGL2 (Ctrl) and TdIF1 siRNA (siTdIF1)-transfected H1299 and H1975 cells were measured via ChIP-qPCR, as described in (Figure 4C). (**B**) The H3K4me3 level at the E-cadherin promoter region. ChIP assays were performed as described in (**A**) with anti-H3K4me3 antibody. (**C**) LSD1 is essential for TdIF1-initiated transcriptional repression. Enrichment at the E-cadherin promoter was measured through a ChIP-qPCR assay in untransfected (Ctrl), sp2509-treated, Flag-TdIF1-transfected, and Flag-TdIF1 + sp2509-treated H1299 cells. (**D**) Migration ability in H1299 cells treated with LSD1 inhibitor, TdIF1 overexpression vector, or both. H1299 cells were treated with Flag-TdIF1 and LSD1 inhibitor sp2509. The migration ability of H1299 cells was measured as described in Figure 2. Data are shown as mean ± SD. * *p* < 0.05, ** *p* < 0.01, *** *p* < 0.001.

As LSD1 is capable of demethylating active H3K4me1 and H3K4me2, it is conceivable that LSD1 may contribute to the TdIF1-mediated transcriptional repression of E-cadherin. We performed ChIP analysis to assess potential changes to active H3K4 methylation at the E-cadherin promoter regulated by TdIF1. Using an antibody specific for H3K4me2, we detected relatively low levels of H3K4me2 at the promoter of the E-cadherin gene in the H1299 and H1975 cells, and a significant increase of H3K4me2 specifically at the E-cadherin promoter region in the siTdIF1-transfected H1299 and H1975 cells (Figure 5A). The levels of H3K4me2 in the E-cadherin promoter correlated with E-cadherin transcription. Moreover, when we performed the ChIP assay to assess the abundance of tri-methylated H3K4 (H3K4me3) at the E-cadherin promoter, there was no significant difference in H3K4me3 levels between the control and TdIF1-KD cells (Figure 5B). These data suggest that TdIF1 regulates H3K4me2, but not H3K4me3, at the E-cadherin promoter.

We next investigated whether TdIF1 recruits LSD1, which subsequently demethylates H3K4me2. We conducted rescue assays in a ChIP assay using H1299 cells that had been transfected with Flag-TdIF1. First, we observed that the downregulation of TdIF1 following the siTdIF1 treatment of NSCLC cells reduced LSD1 (Figure 5A) and increased H3K4me2 (Figure 5A,B) at the E-cadherin promoter. Conversely, the transfection of Flag-TdIF1 to overexpress TdIF1 increased LSD1 and reduced H3K4me2 at that promoter (Figure 5C). When NSCLC cells were treated with the LSD1 inhibitor sp2509, both H3K4me2 and RpolII were enriched at the E-cadherin promoter, as expected, suggesting that TdIF1 acts by recruiting LSD1, which in accordance with our observation that RpolII was enriched after treatment with siTdIF1 (Figure 4D). Furthermore, LSD1 inhibition with sp2509 reduced LSD1 recruitment to the E-cadherin promoter, while the overexpression of Flag-TdIF1 increased it (Figure 5C). The combined treatment with sp2509 and Flag-TdIF1 partly rescued the sp2509-mediated reduction, and the Flag-TdIF1-mediated enrichment, in LSD1 at the promoter. The combined treatment similarly partly rescued the sp2509-mediated enrichment, and the Flag-TdIF1-mediated reduction, in H3K4me2 and RpolII at the promoter (Figure 5C). Furthermore, we confirmed that there were functional changes in cell migration through the wound-healing assay. The results showed that the LSD1 inhibitor could partially rescue TdIF1-OE-induced migration in H1299 cells (Figure 5D). These data revealed that LSD1 is essential for the TdIF1-mediated transcriptional repression of E-cadherin.

### 2.6. TdIF1 Knockdown Synergizes LSD1 Inhibitors in the Suppression of the Metastatic Cell Invasion of NSCLC

**Figure 6:**
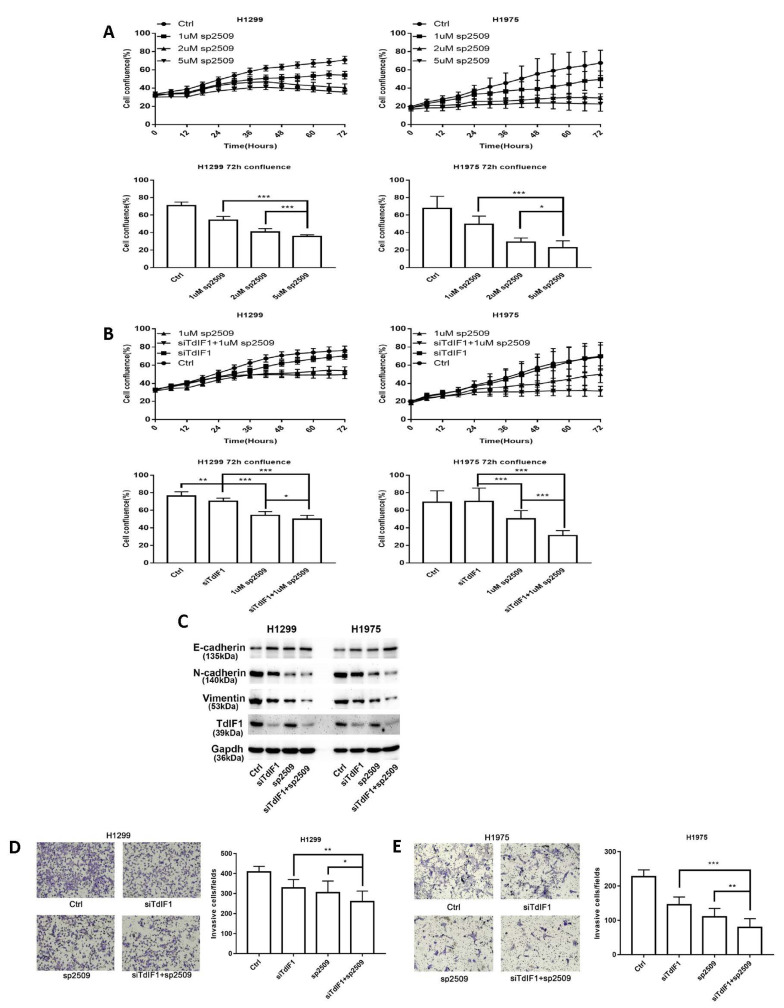
The combination effect of LSD1 inhibitors with siTdIF1 in NSCLC cells. (**A**,**B**) Cell growth of LSD1 inhibitors and siTdIF1-treated cells. Cells were treated with an LSD1 inhibitor or siTdIF1. Cell confluence (%) after treatment with different concentrations LSD1 inhibitor is shown (**A**). Cell confluence (%) of the LSD1 inhibitor and siTdIF1 alone and in combination are also shown (**B**). (**C**) Levels of EMT-associated proteins in cells treated with siTdIF1 or LSD1 inhibitor. Western blot analysis of the protein levels in treated H1299 and H1975 cells. (**D**,**E**) Invasion ability of cells treated with LSD1 inhibitor or siTdIF1, alone and in combination. The invasion abilities of treated H1299 (**D**) and H1975 (**E**) cells were measured as described in Figure 2E. Data are shown as mean ± SD. * *p* < 0.05, ** *p* < 0.01, *** *p* < 0.001.

To investigate the combined effect of LSD1 inhibitors with siTdIF1, we first studied the effect of LSD1 inhibitor sp2509 as a single agent in NSCLC cells. The results showed that the LSD1 inhibitor induced a concentration-dependent cell growth repression in NSCLC cell lines, detected by the measurement of % cell confluence after treatment for 72 h (Figure 6A). Based on these results, we further studied the combined effect of sp2509 with siTdIF1. The NSCLC cell lines were treated with sp2509 and siTdIF1 for 72 h, individually or in combination. The % cell confluency was assessed using an Incucyte Zoom live cell imaging system. The confluency of the cells treated with a combination of sp2509 and siTdIF1 was much lower than those in cells treated with each individually (Figure 6B).

Cell invasion is an indicator of cancer metastasis in NSCLC. To further validate the TdIF1-LSD1 interaction and to explore the potential additive and/or synergistic suppressive effect of tumor cell invasion by simultaneously repressing both molecules, we measured the expression of E-cadherin, N-cadherin, and vimentin using Western blot analysis. The upregulation of E-cadherin and downregulation of N-cadherin and vimentin were greater after combined treatment with the LSD1 inhibitor sp2509 and siTdIF1 than after treatment with either agent alone (Figure 6C). We also measured the invasion capacity of the cells after treatment. Consistent with the changes in cadherin, treatment with siTdIF1 or sp2509 repressed invasion capacity, while the combination of both siTdIF1 and sp2509 did so to a greater degree than either agent alone, in both H1299 cells (Figure 6D) and H1975 cells (Figure 6E). These data indicated that a combination of siTdIF1 with the histone demethylase LSD1 inhibitor significantly suppressed tumor cell growth and invasion, highlighting the potential of combining agents to maximize the biological effects, including the suppression of tumor metastasis, in NSCLC.

## 3. Discussion

The dysregulation of EMT is emerging as a novel theme in tumor biology. However, our understanding of how EMT contributes to the initiation and progression of malignancy (including tumor metastasis) remains incomplete. Here we report a previously unknown association between potential transcription factor TdIF1 expression and altered demethylase LSD1 activity during metastasis and the malignant transformation of NSCLC cells.

Little is known about the relationship between TdIF1 and tumor progression at the present time; instead, the majority of studies have focused on the role of TdIF1 as a potential transcription factor/coactivator. As a transcription factor, TdIF1 recognizes AT tracts in DNA and binds to the 5’-TGCATG-3’ sequence to activate RAB20 transcription [16,17]. Researchers have found that TdIF1 upregulates Myh4 and reduces Myh7 promoter activity, thereby promoting muscle fiber switch and muscle hypertrophy [18,19]. Recent studies have shown that TdIF1 in the nucleosome can also bind HDAC1/2 and TRERF1 (TRep-132) as a subunit to participate in the formation of a new mitogenic deacetylase complex (MiDAC) [20], and specifically binds to cyclin A [21]. In addition to being involved in cell cycle regulation, MiDAC also plays a vital role in neurite development and chromosome alignment during mitosis in cancer cell lines [22,23]. It has been reported that the TdIF1-HDAC interaction promotes tumor growth through the deacetylation of p53, and thus TdIF1 might be a key therapeutic target in oral squamous cell carcinomas [24]. Our previous study also found that TdIF1 is highly expressed in NSCLC patients and is associated with a poor prognosis. Furthermore, we deduced the signal pathways that TdIF1 may participate in and potential target molecules through bioinformatic analysis and the direct inhibition of potential targets [10]. However, the mechanisms responsible for that participation and therapeutic targeting potential remain unclear. In this study, we found that the high expression of TdIF1 is highly correlated with the TNM staging of LUAD patients (Figure 1) and elucidated a novel mechanism by which TdIF1 serves as a transcriptional corepressor to regulate gene expression and promote NSCLC metastasis through LSD1.

NSCLC accounts for more than 80% of all lung cancers. In recent years, however, it has been found that the histological type of NSCLC has changed in certain countries and regions: lung squamous cell carcinoma (LUSC, which was the predominant histotype) has decreased, while lung adenocarcinoma (LUAD) is still increasing in both males and females [25]. In addition to traditional surgical resection, radiotherapy, and chemotherapy, personalized medicine that targets specific genes, such as EGFR and ALK, have also been used in the treatment of metastatic NSCLC. Regardless, the overall survival rate of patients remains low [4,26]. NSCLC patients with poor prognosis (for example, with metastatic and/or drug-resistant disease) have tumors associated with acquired EMT [27]. Through RNA interference experiments in three lung adenocarcinoma cells, we found that the knockdown of TdIF1 upregulates the expression level of E-cadherin while downregulating the expression of N-cadherin and vimentin (Figure 2). Moreover, migration and invasion were inhibited in NSCLC cells with knocked down TdIF1. These results suggest that TdIF1 is involved in a novel mechanism of EMT regulation in NSCLC, further confirmed in a tumor xenograft model in nude mice (Figure 3).

LSD1 has been found to be highly expressed in liver cancer, lung cancer, breast cancer, and prostate cancer, and promotes the growth and invasion of cancer cells by participating in a variety of chromatin epigenetic modifications [14,28,29,30]. Despite the well-known H3K4me demethylating effect of LSD1, it has also been reported that LSD1 induces the demethylation of repressive H3K9me2 and interacts with another transcription factor (GATA2) to form a complex. In addition, it promotes RNA polymerase II recruitment and leads to the activation of gene transcription [31]. Researchers also found that LSD1 is involved in the regulation of EMT. The zinc finger transcription repressor Snail, which serves as the central mediator of EMT and the direct repressor of E-cadherin, has a SNAG domain similar to the histone H3 tail and functions as a molecular hook to recruit LSD1 to repress gene expression in metastasis [32,33]. Notably, the acetylation of LSD1 has been reported to reduce its association with nucleosomes, thus increasing histone H3K4 methylation at its target genes and activating transcription [34]. In a study of triple-negative breast cancer (TNBC) it was found that LSD1 inhibitors and the transcription repressor Slug (a Snail family member) blocked the Slug-mediated repression of the E-cadherin promoter and inhibited tumor cell motility and invasion without any effect on proliferation [35]. Blocking Slug activity suppressed the metastatic spread of TNBC and inhibited the tumor colonization of the bone [36]. Not only that, but also for prostate cancer, neuroblastoma, and endometriosis, LSD1 shows potential as a therapeutic target [37,38,39]. In addition, LSD1 can also play an important role in the development of many diseases through the action of a variety of lncRNAs or miRNAs [40,41]. Recent studies have found that the depletion of LSD1 can enhance anti-tumor immunity and enable checkpoint blockades, suggesting that LSD1 inhibition might be an effective adjuvant treatment with immunotherapy for poorly immunogenic tumors [42,43,44]. In support of these hypotheses, the data shown in this study reveals that TdIF1 interacts with LSD1, binds to the E-cadherin promoter, and regulates EMT as well as cell migration and invasion in NSCLC cells. Moreover, the LSD1 inhibitor impairs TdIF1-induced EMT (Figure 4 and Figure 5).

Because existing treatment options have no substantial impact on the 5-year overall survival rate of NSCLC patients, researchers are actively seeking new and more effective treatments [4]. The efficacy of epigenetic drugs alone in the treatment of tumors has been relatively disappointing, and a current research focus is on a combined therapy of epigenetic drugs with chemotherapy, radiotherapy, immunotherapy, and targeted therapy [6,15]. Many LSD1 inhibitors are currently in clinical trials [45]. Researchers have found that combining EGFR TKIs and LSD1 inhibitors had a better therapeutic effect in NSCLC and could effectively inhibit the appearance of drug resistance [46]. In this study, we report that TdIF1 interacts with LSD1 and recruits LSD1 to the E-cadherin promoter region to regulate its transcriptional activity, thereby promoting EMT and tumor metastasis. LSD1 is essential for TdIF1-mediated transcriptional repression of E-cadherin. We demonstrate that the knockdown of TdIF1 combined with LSD1 inhibitors showed better anti-migration and invasion ability in NSCLC cells than a single agent, as well as the regulation of EMT related factors (Figure 6).

Taken together, our results indicate that TdIF1 plays an important role in promoting the development of NSCLC and is a potential prognostic biomarker for NSCLC. Therefore, targeting TdIF1 and combining it with demethylase inhibitors might be a novel therapeutic strategy for NSCLC treatment.

## 4. Materials and Methods

### 4.1. Plasmid, siRNA, and LSD1 Inhibitor

The pcDNA3.1-Flag-TdIF1, pCDH-HA-LSD1 and pLKO.1-puro-shTdIF1 constructs were generated by Focus Biology (Nanchang, Jiangxi, China). The small interfering RNAs (siRNAs), both targeting TdIF1 (siTdIF1) and non-targeting control (siGL2), were purchased from Dharmacon (Lafayette, CO, USA). The target sequence of siTdIF1 was 5′-GAAAGUAUAUGGAGACACU-3′. The LSD1 inhibitor SP-2509 (EMD Millipore, Billerica, MA, USA) was diluted with DMSO to the indicated concentrations.

### 4.2. Data Analysis Using the Cancer Genome Atlas (TCGA)

Both gene expression data and clinical data, including tumor stage information from LUAD (lung adenocarcinoma) and LUSC (lung squamous cell carcinoma) patients, were downloaded from TCGA Data Portal (https://portal.gdc.cancer.gov/, 12 October 2021). All data are available online, and this present study meets the publication guidelines provided by TCGA (http://cancergenome.nih.gov/publications/publicationguidelines, 12 October 2021). The analysis of the TdIF1 expression profiles in LUAD patient tumor tissues (*n* = 526) was compared with adjacent non-tumor lung tissue (*n* = 59); in LUSC patients, tumor tissues (*n* = 501) were compared with adjacent non-tumor lung tissue (*n* = 49). Based on these data, further analysis was performed with respect to tumor stage (lymph node metastasis) in LUAD/LUSC patient tissues and adjacent non-tumor lung tissues.

### 4.3. Animals

BALB/c nude mice were purchased from Changsha Laboratory Animal Co. Ltd.(Changsha, China) and housed in an SPF (specific pathogen-free) animal center. The use of all mice in this study complied with the Regulations for the Administration of Affairs Concerning Experimental Animals of China and the ethical approval of the institutional Animal Care and Use Committee of Nanchang University, China.

### 4.4. Cell Culture

The human lung adenocarcinoma cell lines A549, H1299, and H1975 were obtained from the American Type Culture Collection (ATCC) and cultured with RPMI-1640 medium (Invitrogen, Carlsbad, CA, USA) at 37 °C, with a CO_2_ concentration of 5% and an FBS concentration of 10%. The culture method was in accordance with ATCC cell culture standards.

### 4.5. Transfection and Interference Assay

EndoFectin (GeneCopoeia, Rockville, MD, USA) was used for the cell transfection and the siRNA interference assay according to the manufacturer’s instructions. Briefly, the cells were grown to 50% confluence for siRNA treatment and above 80% for overexpression analysis. Plasmids or siRNAs were incubated with a transfection reagent in Opti-MEM for 20 min at 20 °C before addition to the medium. Then, the cells were cultured in a transfection medium and harvested at 48 h and 72 h.

### 4.6. Wound Healing Assay

NSCLC cells were seeded in a six-well plate to form a confluent monolayer. A pipette tip was then used to scrape the single layer in a straight line to create scratches or wounds. The cells were cultured in RPMI 1640 without FBS and imaged using a phase-contrast microscope. Wound areas were quantified using ImageJ software (version 1.6.0-20, National Institutes of Health, Bethesda, MD, USA).

### 4.7. Cell Migration and Invasion Assay

Cultured NSCLC cells dispersed into single cells via trypsin treatment and were resuspended in a serum-free medium at 3–6 × 10^4^ cells per 100 μL. For cell migration assays, cells were placed in a 24-well transwell and 600 μL of medium containing 20% FBS was added to the lower chamber. Invasion assays were performed in transwell plates precoated with a Matrigel membrane matrix according to the manufacturer’s protocol. The cells were harvested 24 h later and fixed with 4% paraformaldehyde for 20 min. The cells were stained with 0.1% crystal violet followed by counting the cells in three random high-power views under the microscope.

### 4.8. Incucyte Live-Cell Imaging System

The cell growth was imaged using the Incucyte Zoom live cell imaging system (Essen Bioscience, Ann Arbor, MI, USA). Cells were scanned once per hour from 0 to 72 h post-treatment. The % of cell confluence was calculated using acquired phase-contrast images and Incucyte Zoom software (version 2018A, Essen Bioscience, Ann Arbor, MI, USA).

### 4.9. Quantitative Reverse Transcription-Polymerase Chain Reaction (qRT-PCR)

Total RNA was extracted from tissues or cultured cells using the TRIzol reagent (Invitrogen, Carlsbad, CA, USA) according to the manufacturer’s instructions. A M-MuLV Reverse Transcriptase Kit (New England BioLabs, Ipswich, MA, USA) was used for cDNA synthesis. The qPCR was performed using a SensiFAST SYBR No-ROX One-Step kit (Bioline, Tauton, MA, USA) on the CFX96™ real-time PCR detection system (BioRad, Mississauga, ON, CA, USA). The relative quantification of mRNA expression was represented by 2^-ΔΔCT^. The primers for TdIF1 were: 5′- ACTGAACGTGCGAGACAATGT -3′(forward) and 5′- GCTCATGGGTCAATCTGGGTATT -3′(reverse). The primers for E-cadherin were: 5′-CGAGAGCTACACGTTCACGG-3′(forward) and 5′-GGGTGTCGAGGGAAAAATAGG-3′(reverse). The primers for N-cadherin were: 5′- AGCCAACCTTAACTGAGGAGT-3′(forward) and 5′- GGCAAGTTGATTGGAGGGATG-3′(reverse). The primers for Vimentin were: 5′- GGACCAGCTAACCAACGACA-3′(forward) and 5′- AAGGTCAAGACGTGCCAGAG -3′(reverse). The primers for GAPDH were: 5′- TGACTTCAACAGCGACACCCA -3′(forward) and 5′- CACCCTGTTGCTGTAGCCAAA -3′(reverse).

### 4.10. Western Blot Analysis

Cells and tissues were harvested and lysed with a RIPA buffer for protein extraction. BCA kits were used to determine the protein concentration. The protein was transferred to 0.22 μm PVDF membrane after being electrophoresed on 10% SDS-Page gel. The membranes were first blocked using 5% (*w*/*v*) skim milk at room temperature, followed by incubation with the primary antibodies against TdIF1 (Abcam, Branford, CT, USA), and E-cadherin, N-cadherin, and vimentin (Invitrogen, Carlsbad, CA, USA), or incubated with an antibody against GAPDH (Proteintech, Wuhan, Hubei, China), at 4 °C overnight. The membranes were incubated with horseradish peroxidase for 1 h. Protein bands were detected after incubation with an ECL chromogenic substrate and analyzed using ImageJ software.

### 4.11. Co-Immunoprecipitation (Co-IP)

Cells were harvested, and protein was extracted with 1 mL of lysis buffer containing a protease inhibitor cocktail (Roche, Mannheim, Germany). The protein was mixed with 20 μg antibodies against TdIF1 (Abcam, Branford, CT, USA), Flag, HA, and a negative control IgG (Cell Signaling Technology, Danvers, MA, USA), respectively, at 4 °C overnight. Then, 50 μL Protein G Agarose (Roche, Mannheim, Germany) was added to each protein/Ab mixture and incubated for 3 h at 4 °C, followed by washing with lysis buffer. An SDS sample buffer was added to the immunoprecipitated proteins. Western blotting was conducted to detect Co-IP proteins using antibodies against TdIF1, LSD1, Flag, and HA (Cell Signaling Technology, Danvers, MA, USA).

### 4.12. Chromatin Immunoprecipitation Assay (ChIP)

A ChIP assay was performed using a SimpleChIP Plus Enzymatic Chromatin IP Kit (Cell Signaling Technology, Danvers, MA, USA) in accordance with the manufacturer’s instructions. Briefly, cells were fixed with 1% formaldehyde for 10 min and then disrupted in SDS lysis buffer. Chromatin was sonicated to shear the DNA into fragments with an average length of 100–200 bp, as verified using agarose gel electrophoresis. Chromatin was then immunoprecipitated with antibodies against TdIF1, LSD1, H3K4m2, H3K4m3, and RpolII (Cell Signaling Technology, Danvers, MA, USA). An equal amount of immunoglobulin G was used as a negative control. The precipitated chromatin was eluted with magnetic beads and purified before being subjected to q-PCR. The primers for the proximal promoter region of the E-cadherin gene used for ChIP-qPCR were: 5′- GCCCTTTCTGATCCCAGGTC-3′ (forward) and 5′- TAGCCTGGAGTTGCTAGGGT-3′ (reverse).

### 4.13. Human Lung Cancer Xenograft Model

For this process, 2 × 10^6^ H1975 cells were resuspended in PBS (200 μL) and injected subcutaneously into 5-week-old female BALB/c-nu mice. The tumor sizes were measured every three days using a vernier caliper. Tumor volumes were calculated using the formula V = (L × W^2^)/2.

### 4.14. Human Tissue Samples

Human *tissue* specimens were collected and stored at the London Health Sciences Centre. The protocol was approved by the Institutional Review Board, and the studies were conducted in accordance with the ethical guidelines of the Declaration of Helsinki. Written informed consent was obtained from patients.

### 4.15. Statistics

Data are presented as the mean ± SD from experiments performed at least three times. Differences between the two groups were assessed with Student’s *t*-test or a one-way ANOVA. Differences were considered significant if *p*-values were less than 0.05.

## Data Availability

All images and data available on reasonable request. All other data are available from the corresponding authors.

## Abbreviations

TdT: terminal deoxynucleotidyltransferase; TdIF1, TdT-interacting factor 1; NSCLC, non-small cell lung cancer; TCGA, The Cancer Genome Atlas; LSD1, lysine-specific demethylase 1; EMT, epithelial–mesenchymal transition.
